# Potent and Specific Antibacterial Activity against *Escherichia coli* O157:H7 and Methicillin Resistant *Staphylococcus aureus* (MRSA) of G17 and G19 Peptides Encapsulated into Poly-Lactic-Co-Glycolic Acid (PLGA) Nanoparticles

**DOI:** 10.3390/antibiotics9070384

**Published:** 2020-07-07

**Authors:** Nicolás Gómez-Sequeda, Jennifer Ruiz, Claudia Ortiz, Mauricio Urquiza, Rodrigo Torres

**Affiliations:** 1 Grupo de Investigación en Bioquímica y Microbiología (GIBIM), Universidad Industrial de Santander, Bucaramanga 680002, Colombia; nicosebasemi@gmail.com (N.G.-S.); jennifer.ruiz@correo.uis.edu.co (J.R.); 2 Escuela de Microbiología, Facultad de Salud, Universidad Industrial de Santander, Bucaramanga 680002, Colombia; 3 Departamento de Química, Universidad Nacional de Colombia, Bogotá 111321, Colombia; mauriciourquiza@yahoo.com; 4 Departamento de Ciencias Básicas, Facultad de Salud, Universidad Industrial de Santander, Bucaramanga 680002, Colombia; rodrigo.torres@ecopetrol.com.co

**Keywords:** nanoencapsulation, antimicrobial peptides, MRSA, *E. coli* O157:H7

## Abstract

Antimicrobial peptides constitute an excellent alternative against conventional antibiotics because of their potent antimicrobial spectrum, unspecific action mechanism and low capacity to produce antibiotic resistance. However, a potential use of these biological molecules as therapeutic agents is threatened by their low stability and susceptibility to proteases. In order to overcome these limitations, encapsulation in biocompatible polymers as poly-lactic-glycolic-acid (PLGA) is a promising alternative for increasing their stability and bioavailability. In this work, the effect of new synthetic antimicrobial peptides GIBIM-P5S9K (G17) and GAM019 (G19) encapsulated on PLGA and acting against methicillin resistant *Staphylococus aureus* (MRSA) and *Escherichia coli* O157:H7 was studied. PLGA encapsulation allowed us to load around 7 µg AMPs/mg PLGA with an efficiency of 90.5%, capsule sizes around 290 nm and positive charges. Encapsulation improved antimicrobial activity, decreasing MIC50 from 1.5 to 0.2 (G17NP) and 0.7 (G19NP) µM against MRSA, and from 12.5 to 3.13 µM for *E. coli* O157:H7. Peptide loaded nanoparticles could be a bacteriostatic drug with potential application to treat these bacterial *E. coli* O157:H7 and MRSA infections, with a slow and gradual release.

## 1. Introduction

Using a broad spectrum of antibiotics has led to the emergence of multidrug resistant (MDR) microorganisms, intensifying public health problems such as bacterial infections with the consequent spreading in the population. The World Health Organization (WHO) includes methicillin resistant *Staphylococcus aureus* (MRSA) and enterohemorrhagic *Escherichia coli* as high and critical risk pathogens, which are included in the report "Global priority list of antibiotic resistant bacteria for research, discovery and development of new antibiotics" WHO [1,2].These pathogens are responsible for the majority of nosocomial and community-associated infections, occupying the first and second place among the most common drug-resistant pathogens with 15 and 12% of the total cases, respectively. MRSA and *E. coli* O157:H7 present multiple mechanisms of antibiotic resistance, making the infections associated to these pathogens difficult to treat. Recently, there has been the emergence of ‘super resistant bugs’, multidrug-resistant forms with an increased level of resistance or those resistant to multiple antibiotics such as MRSA, which is estimated to cause over 10,600 deaths per year [3]. *E. coli* O157:H7 is the most important Shiga toxin-producing *E. coli* (STEC) serotype in relation to public health, being responsible for over 63,000 illnesses per year and an annual cost of 405 million dollars, related to productivity, medical care, and premature deaths [4,5].

In this sense, antimicrobial peptides (AMPs) have emerged as new antimicrobial compounds acting over a broad variety of both conventional and new microbial targets, and they represent a promising therapeutic option against proliferation of antibiotic-resistant pathogens [6,7]. In addition, these AMPs are effector molecules of the innate immune system; usually cationic (positive net charge between +2 and +9 at physiological pH) with sizes lower than 10 kDa. AMPs differ from most antibiotics due to their multiple mechanisms of action and broad action spectrum [8]. Among the advantages that AMPs can offer are: (I) wide antimicrobial activity against several microorganisms such as Gram negative and Gram positive bacteria, fungi and viruses [9]; (II) their both rapid and direct action allows them to kill microorganisms mediated by bacterial membrane destabilization [10]; (III) lipid bilayer structures of bacterial membranes restrict antimicrobial resistance against AMPs [11].

However, therapeutic use of AMPs is restricted because their susceptibility to degradation or inactivation by pH changes, protease activity or high ionic strength [10]. Moreover, AMPs can display low stability in blood plasma [12] and high hemolytic activity [13]. Therefore, nanoencapsulation of bioactive compounds in biodegradable polymers has been explored in order to enable its therapeutic use [14]. These biopolymers offer multiple advantages; they can protect active ingredients against both chemical and enzymatic degradation, site specific delivery, protection, controlled release, enhanced therapeutic efficiency and reduction of side effects [14]. Encapsulation in polymeric nanoparticles is one of the most promising strategies for improving AMP stability and delivery. PLGA nanoparticles have been utilized as a drug delivery system due to their good biocompatibility and adjustable biodegradability [15,16].

For instance, GIBIM-P5S9K (G17), a new antimicrobial peptide, designed through a genetic algorithm optimization strategy, has showed a wide range of activity against MRSA, *E. coli* O157:H7 and *P. aeruginosa*. In addition, previous studies have shown that a nanoencapsulated preparation of G17 displays both low erythrocyte hemolysis and cytotoxicity, and it is stable in human sera [17]. In this work, GAM019 (G19), an analogue of the G17 peptide was synthesized by solid-phase chemistry and encapsulated in PLGA at different peptide/biopolymer ratios in order to obtain the best physicochemical properties of surface charge, size, encapsulation efficiency and also improve its antimicrobial activity against MRSA and *E. coli* O157:H7.

## 2. Results and Discussion

### 2.1. Peptide Synthesis and Characterization

In this work, a new peptide sequence (G19) was generated by changing Gly by Ala and Phe by Trp in the G17 peptide sequence in order to increase the alpha-helix stability of this peptide and the aromatic interaction (Table 1). The G17 peptide was previously designed by using a genetic algorithm optimization strategy, using the CAMP database and physicochemical descriptors such as isoelectric point, charge, hydrophobicity and instability index; this peptide has a high antimicrobial activity previously demonstrated against *E. coli* O157:H7 and MRSA [18]. Using the AMP prediction software in the CAMP_R3_ web pages [19], the G19 peptide sequence exhibited a 97.3% probability of being antimicrobial, with an instability index of 24.68, indicating that the peptide has a good stability [20], and a GRAVY value in which G19 (0.735) is higher than G17 (0.382) (Table 1). Both peptides, G17 and G19, were synthesized via the solid-phase method and purified by RP-HPLC; peptides G17 and G19 presented a molecular mass in silico of 1804 and 1864 Da respectively, which was corroborated by MALDI-TOF experimentally (1804.71 and 1864.12 KDa, respectively; see Supplementary Materials File Figure S1). G19 showed an alpha helix in its secondary structure as determined by circular dichroism, similar to G17 (Supplementary Materials File Figure S2).

According to the current model of AMP-interaction with the cell membrane, there are at least two interacting regions in these peptides; a positive charged region and a hydrophobic one. Both G17 and G19 are able to form amphipathic helices in vitro according to the CD spectra (Supplementary Materials File Figure S2), with at least two regions; one hydrophobic and another positive domain. The positive domain in peptide G17 is higher than peptide G19, because G17 contains an additional Lys residue in the G17 sequence. Changes of Phe and Lys in the position 8 and 9 present in peptide G17 by Trp and Ser in peptide G19 could be responsible for the differences in the specificity of both peptides (Table 1). The hydrophobic region of the peptides G19 and G17 favors the interaction with the cell membranes, increasing the probability for peptides G17 and G19 to be more antibacterial and potentially hemolytic [18,21]. Encapsulation is an excellent strategy that is able to minimize possibly undesirable characteristics of synthetic AMPs [22]. For this reason, both peptides were encapsulated into PLGA nanoparticles in order to improve their interaction with the bacterial membrane before the peptide is released, and at the same time decrease their cytotoxicity, reduce degradation and enhance peptide bioavailability in sites of bacterial infection [18,23,24].

### 2.2. Physicochemical Properties of AMP-NPs 

In nanoparticle systems, the size and surface charge of the nanoparticle are crucial parameters that affect the releasing rate, encapsulation efficiency, pharmacokinetics and even the antimicrobial activity of the pharmaceuticals. Therefore, it is essential to find the optimal balance between these variables to improve the biological activity of the nanoparticle [25,26]. Several experiments were carried out in order to obtain different AMP/PLGA ratios and determine the best physicochemical properties of the NPs; results are shown in Table 2. In both experiments, a poly dispersion index (PDI) <0.3 was obtained, demonstrating a narrow distribution and a good reproducibility of the method [27].

**Figure 1:**
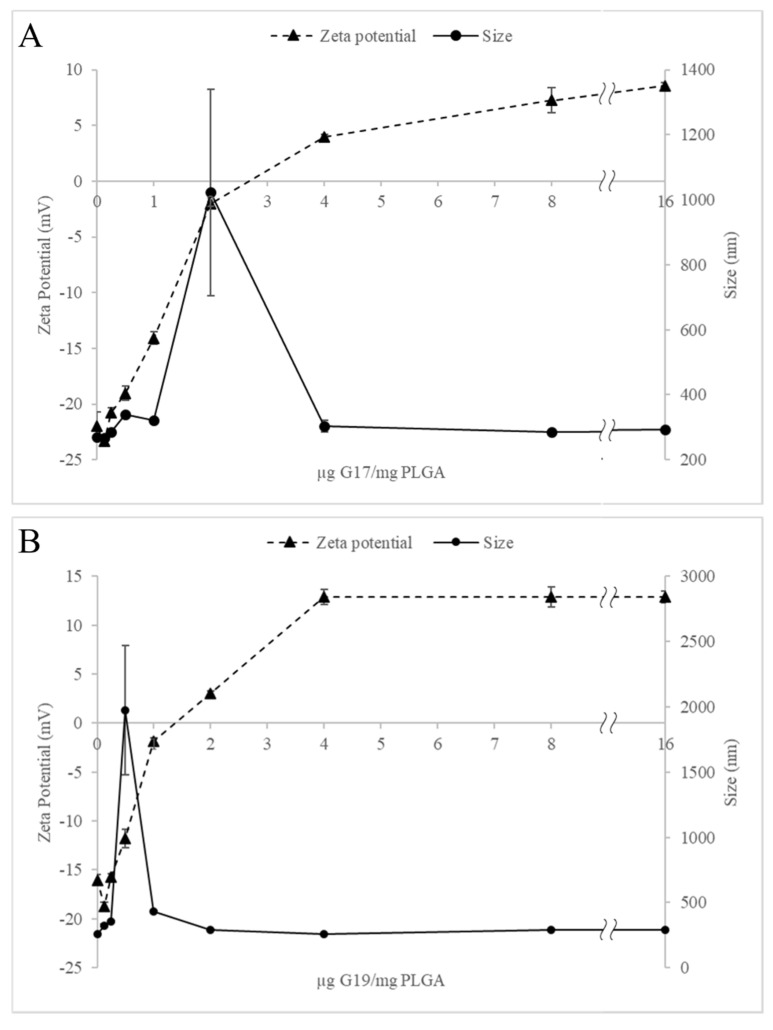
Effect of the peptide/polymer ratio on zeta potential and size of G17NP (**A**) and G19NP (**B**). Data are the mean ± SD (*n* = 3).

It was observed that at 2 μg G17/mg PLGA ratio (mean size = 1022 ± 317.18 nm, zeta potential = −2.06 ± 0.21 mV, Figure 1A) and 0.5 μg G19/mg PLGA ratio (mean size = 1976 ± 496.56 nm, zeta potential = −11.8 ± 0.95 mV, Figure 1B), the zeta potential of the nanoparticle is increased, approaching 0 mV and as a consequence a decrease of the repulsion between the nanoparticles, which generates the biggest agglomerations. However, at concentrations where it was higher than 4 μg of AMP/mg PLGA, the nanoparticles were stabilized, obtaining sizes smaller than 310 nm, positive surface charge and poly dispersion index (PDI) < 0.3, which demonstrate a narrow distribution and a good reproducibility of the method (Table 2, Figure 1A,B) [27].

**Figure 2:**
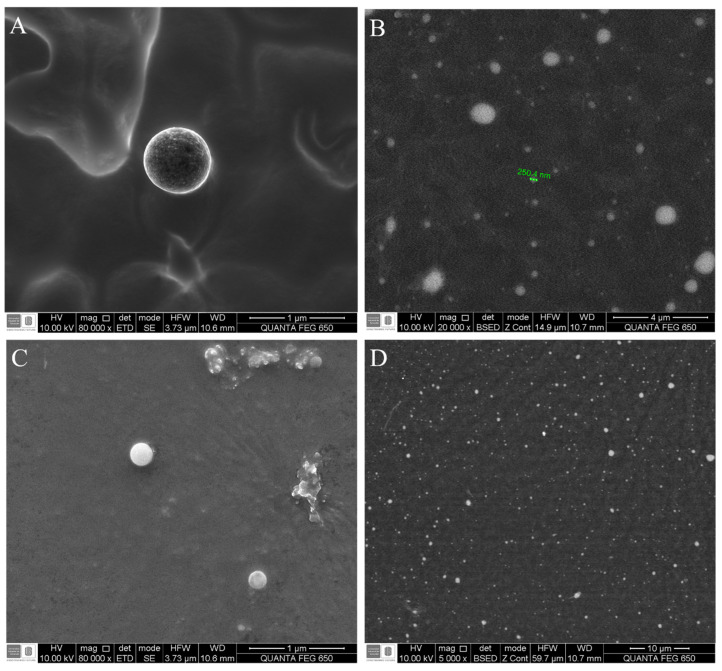
Micrograph of the G19NP and G17NP obtained by scanning electron microscopy (SEM). (**A**) Morphology of the G17NP; (**B**) General shape of the G19NP population; (**C**) Morphology of the G19NP; (**D**) General shape of the G19NP population.

The G17NP shape was perfectly spherical (Figure 2A), which is consistent with the shape of PLGA nanoparticles obtained in previous studies [18]. However, the images with lower magnification showed different populations of nanoparticles, with non NP agglomerations (Figure 2B). On the other hand, the G19NP exhibited a spherical shape with non-agglomerations (Figure 2C), sizes around 290 nm (Figure 2D) and consistent with the hydrodynamic sizes determined by DLS. These nanoparticles were similar not only in size and shape but also dispersion to those obtained by Water et al. 2015 and Cruz et al. 2017 [18,28].

Nanoparticles obtained in this work were comparable in sizes with those obtained for other authors, being, e.g., six times smaller than those obtained by Li et al., 2017 [29], using the electrospray technique, but between 11% and 23% smaller using double emulsion techniques [18,28]. Nevertheless, our nanoparticles encapsulated more peptide (up to 35%) compared to the nanoparticles obtained by Cruz et al. 2017 [18] and with similar percentages of encapsulation (greater than 90%) to those reported in other studies [28,29]. It has been proved that the cationic nanoparticles efficiently interact with the anionic lipids of bacterial membranes [18,30]. In this work, it was possible to obtain cationic nanoparticles (with zeta potential for G17NP = 7.26 mV and G19NP = 12.87 mV) without using cationic polymers such as polyethylenimine or chitosan for their coating, since these polymers have shown cytotoxic activity against eukaryotic cells [31,32].

### 2.3. Encapsulation Efficiency and Load Capacity of the G17NP and G19NP

**Figure 3:**
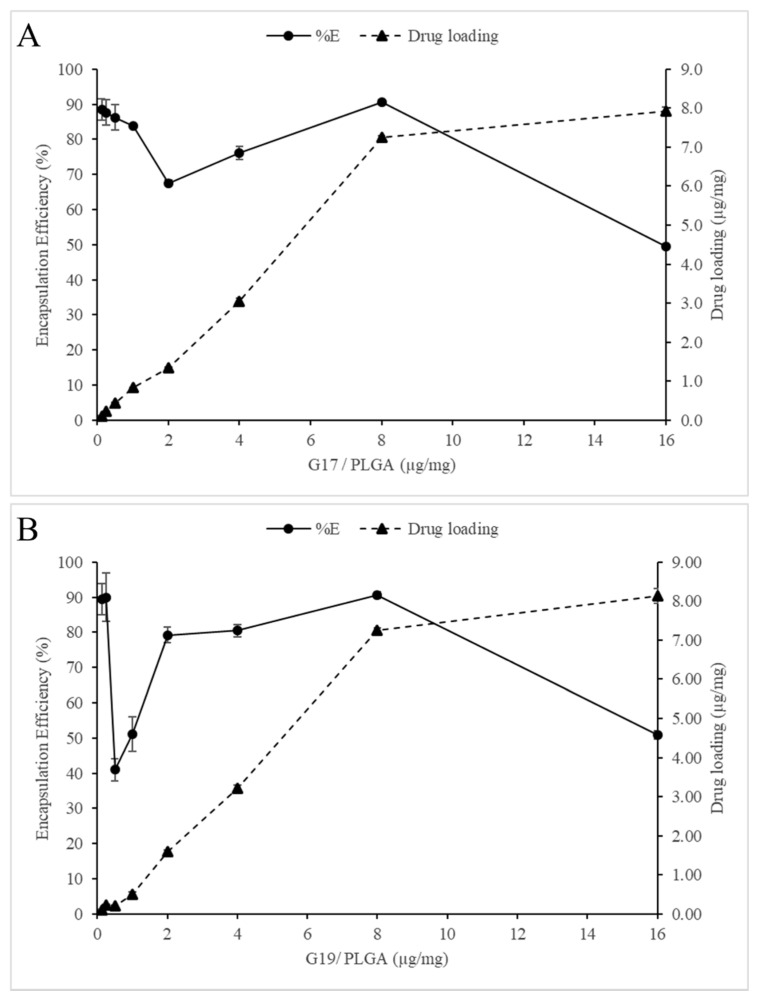
(**A**) and (**B**) shows the influence of the peptide/polymer ratio on drug loading and encapsulation efficiency in PLGA nanoparticles. Data are the mean ± SD (*n* = 4).

The effect of the AMP/PLGA ratio on encapsulation efficiency and peptide loading was analyzed (Figure 3); it was found that AMP-NP with either negative or positive zeta potential values is possible to avoid agglomeration, and a high encapsulation efficiency and peptide loading can be obtained (around 90%). On the other hand, at the ratio of 2 μg G17/mg PLGA and 0.5 μg G 19/mg PLGA, a low encapsulation efficiency was obtained (67 and 41%, respectively). At these ratios, the zeta potential of the nanoparticles approaches 0 mV, generating high instability of the AMP-NP. In this way, the effect of the zeta potential on the NP size and encapsulation efficiency showed similar results to those previously reported by other authors [33,34].

Finally, at the rate of 16 µg/mg of PLGA, although it was also the rate with the lowest encapsulation efficiency (40.50% G17NP and 50.85% G19NP), it is possible that at this point, the transport system had become saturated, causing an excess of free peptide.

### 2.4. In Vitro Release of Antimicrobial Peptides from PLGA-NP

**Figure 4:**
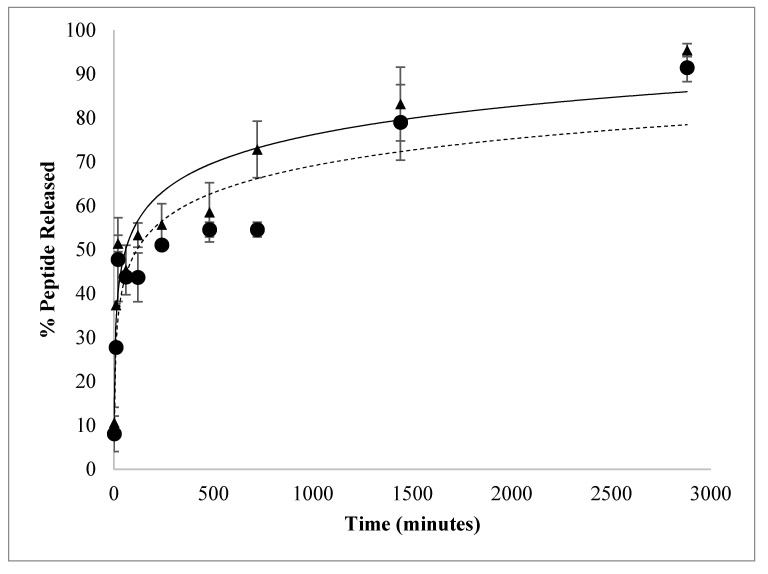
In vitro release of G17NP (▲) and G19NP (●). Release profile of polymeric nanoparticles under the same incubation conditions, 37 °C, pH 7.4 and 50 rpm.

A fresh batch of nanoparticles was synthesized using the best peptide/polymer ratio (8 µg AMP/mg PLGA) for the in vitro peptide releasing profile. More than 44.0 ± 1.1% of G17 and 45.0 ± 1.1% of G19 were released in the first 60 min from these nanoparticles. Subsequently, a controlled release of these peptides was observed up to 2880 min to release most of the content of the nanoparticle (Figure 4). It is possible that a fraction of the total peptide was embedded on the nanoparticle surface, being rapidly dissociated, while the residual peptide remains inside of the nanoparticle and it is slowly released once PLGA is hydrolyzed. This releasing profile was very similar to that obtained by other authors using different active principles encapsulated [18,32,35].

### 2.5. Determination of Minimum Inhibitory Concentration (MIC) and Minimum Bactericidal Concentration (MBC)

**Figure 5:**
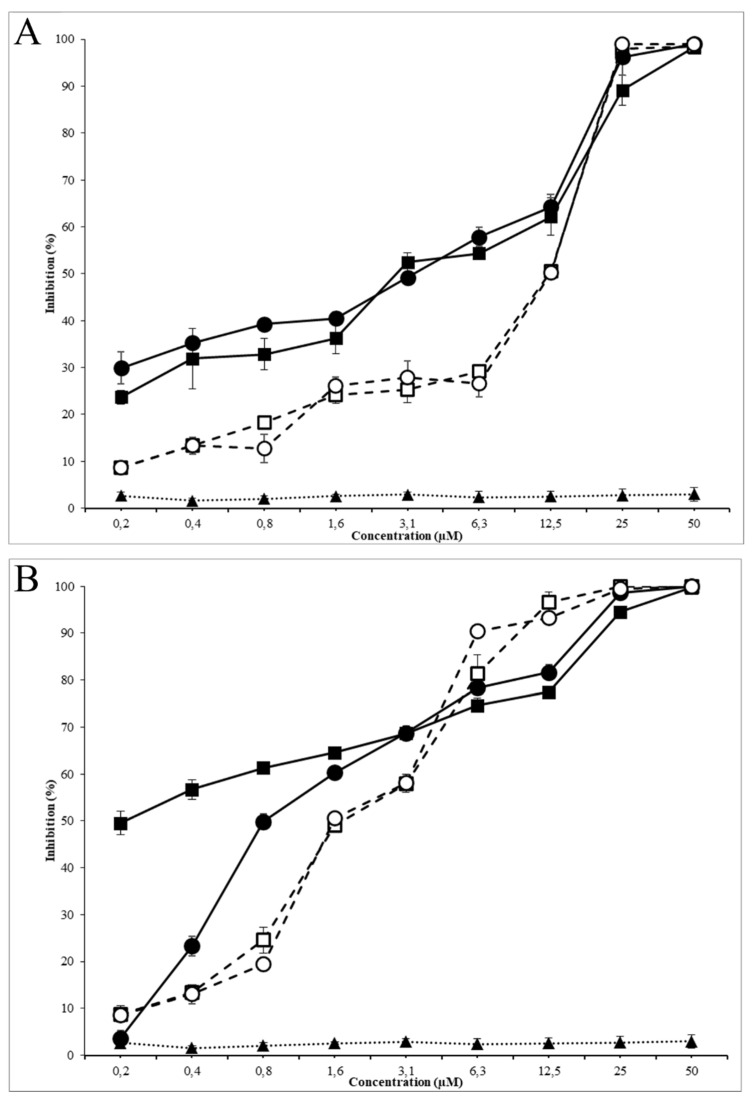
Antimicrobial activity of G19NP, G19 and NP against *E. coli* (**A**) and MRSA (**B**). (■) G17NP, (□) G17, (▲) Empty nanoparticle, (●) G19NP, (○) G19. Data are the mean ± SD (*n* = 3).

Antimicrobial activity assays were performed using nanoparticles of 8 μg AMP/mg PLGA ratio, which presented the highest peptide loading and encapsulation efficiency (Table 2). The growth of *E. coli* O157:H7 and MRSA were evaluated in the presence of free AMPs (G17 and G19), encapsulated AMPs (G17NP and G19NP) and empty nanoparticles (NP) in the range of 0.2 to 100 µM of AMP. Both antimicrobial peptides G17 and G19, showed a very similar growth inhibition activity against *E. coli* O157:H7 and MRSA, with MIC_50_ of 12.5 and 1.5 μM, respectively. On the other hand, peptide nanoencapsulation in PLGA decreased the MIC_50_ up to four times (3.13 μM) for G17NP and G19NP against *E. coli* O157:H7, while against MRSA, the MIC_50_ of the G17NP and G19NP decreased 7 and 2 times, respectively (Table 3). At low peptide concentration, peptide-nanoparticles presented a higher antimicrobial activity than that of the free peptides against both strains; however, the increase in the inhibition along the peptide concentration, in both tested strains, was higher for free peptides than for peptide-nanoparticles. This effect was more remarkable when G17NP was tested against MRSA, which, at 0.2 μM of peptide-NP, was inhibited by around 30% of bacterial growth, while at 100 μM, it obtained an inhibition of <70%. The results of MIC_90_ and MBC also showed a lower increase in the activity of the encapsulated peptide compared to the free peptide activity. Moreover, it was found that G17 and G17NP presented a higher antibacterial activity against *E. coli* and G19 and G19NP showed a higher activity against MRSA. It is possible that the higher antibacterial activity of AMP-NPs compared to AMPs is related to the increase in the focal concentration of the peptide on the bacterial membrane [18]. On the other hand, the lower activity at higher peptide concentrations may be due to the release rate of the AMP from the nanoparticle, since the hydrolysis process of the PLGA can take several hours (Figure 4). It was found that empty nanoparticles (NPs) had no effect on the cell viability of *E. coli* O157:H7 and MRSA (Figure 5).

Peptide G17 presented higher antibacterial activity against *E. coli* O157:H7 than the activity of peptide G19; on the contrary, peptide G19 exhibited higher antibacterial activity against MRSA than peptide G17, with a MIC_50_ of G17 and G19 peptides against *E. coli* O157:H7 and MRSA comparable to the most potent natural peptides such as LL-37, Plectasin or Nisin [36,37,38]. By encapsulating both peptides in PLGA nanoparticles, the antibacterial activity was increased several times. These results were similar to those reported for the encapsulation of other antibiotics to protect them, increasing their antibacterial activity and decreasing their cytotoxicity [28,32].

Moreover, antimicrobial activity of the nanoencapsulated peptides AMP-NP (G17NP and G19NP) against *E. coli O157:H7* and MRSA allowed them to release both peptides in a prolonged way and inhibit the bacterial growth at lower peptide concentrations for a long time. This may be due to the increase in the local concentration of peptides on the bacterial surface, resulting in a higher antimicrobial activity because of electrostatic interactions between cationic nanoparticles and anionic membranes of the Gram-negative and Gram-positive bacteria [39,40].

## 3. Materials and Methods 

### 3.1. Materials

Fmoc-L-amino acids and the Rink-amide 4MBHA resin were purchased from Chempep and Merck Novabiochem, respectively. The reagents used for the encapsulation of the peptide such as PLGA 50:50 (average mol wt 30,000–60,000 Da), the surfactant agent, poloxamer 407 (POL) purified nonionic were purchased from Sigma-Aldrich (USA). All reagents and solvents used for peptide synthesis and nanoencapsulation were of analytical grade. Strains of MRSA and *E. coli* O157:H7 were acquired at the microorganism collection of the Pontificia Universidad Javeriana from Colombia (CMPUJ certified by World Federation of Culture Collection). The maintenance and the microbiological assays were performed in Brain Heart Infusion (BHI) medium, Luria–Bertani (LB) and Müeller–Hinton (MH) media, respectively. All culture media were acquired from MERCK.

### 3.2. Peptide Synthesis and Characterization

Peptides G17 and G19 were synthesized via multiple solid phase peptide synthesis, using substituted rink amide 4MBHA resin (100–200 mesh; loading: 0.63 mmol g^−1^), and following the procedure reported by Houghten [41,42]. The Fmoc group was removed by using piperidine (20% *v/v* in DMF), and each amino acid was coupled using a 4-fold excess of Fmoc-protected amino acid and HOBt/HBTU in the presence of DIPEA for 1 h. Once the synthesis of the amino acid sequence was finished, the peptides were removed from the resin by treatment with tri-fluoroacetic acid (TFA)/tri-isopropyl-silane (TIS)/ethanedithiol/H_2_O for 2 h and the peptides were precipitated with cold diethyl ether [18,43]. The peptides were desalted by gel exclusion chromatography using G-10 columns (Amersham, USA), and purified by reverse phase-high performance liquid chromatography (RP-HPLC) (Jasco Co, Tokyo, Japan) using a Zorbax Eclipse XDB C18 semipreparative column, with water acetonitrile (ACN) gradients containing 5 mM HCl as previously reported by Wadhwani et al. [44]. Finally, the molar mass and secondary structure of purified peptides were determined by MALDI-TOF mass spectrometry and circular dichroism, respectively [45].

### 3.3. Preparation of Polymeric NPs

The AMP encapsulated nanoparticles were synthesized using the double emulsion solvent diffusion method (DES-D) proposed by Cohen-Sela [35] with some modifications. A first emulsion was prepared using a solution of peptide, dissolved in 0.05 M phosphate buffer (pH = 7.4), and dispersing 10% PLGA (*w/v*) in ethyl acetate (EtAc) in a ratio of peptide solution to PLGA dispersion of 2:1. This mixture was homogenized using an ultrasonic processor at 80% of maximum potency (Cole-Parmer 130-Watt Ultrasonic Processors) configured in 2 pulses of 2 s separated by a rest interval of 5 s. The resulting primary emulsion was added to a 0.05 M buffer phosphate (pH = 7.4) containing 20% of Poloxamer 407 in a ratio of buffer/emulsion of 5:1. This mixture was homogenized again using the previously mentioned protocol. Subsequently, EtAc was eliminated by evaporation under reduced pressure using a rotary evaporator (Heidolph Hei-VAP) during 15 min at reduced pressure of 100 mbar. Finally, the nanoparticles were purified using an Amicon Ultra-15 (30,000 MW) ultrafiltration tube by centrifugation at 5000 *g* during 10 min at 4 °C (Thermo Scientific IEC CL31R multispeed) to eliminate the free peptide and the residues from the synthesis. 

### 3.4. Physicochemical Properties of Encapsulated Antimicrobial Peptides (AMP-Nps)

Hydrodynamic size and surface charge of the peptide nanoparticles were determined by dynamic light scattering (DLS) and laser doppler electrophoresis (LDE), using a particle size analyzer based on laser diffraction (Malvern Zetasizer 1000HS, Malvern Instruments, Malvern, UK). A set of experiments was performed varying the amount of peptide from 0.125 μg up to 16 μg for each mg of PLGA in order to determine the possible variations in hydrodynamic size, surface charge and encapsulation efficiency. Results were presented as means of experiments performed in triplicate ± standard deviation. According to the previous results, the best nanoparticles were dried at room temperature and gold coated in order to improve the image quality; these NPs were analyzed by scanning electron microscopy (SEM) using a FEI Quanta 650 microscope to determine the nanoparticle morphology.

### 3.5. Determination of Peptide Loading and Encapsulation Efficiency of AMP-Nps

The peptide encapsulation efficiency in these nanoparticles was determined using both a direct and indirect method. In the direct method, a volume of peptide loaded nanoparticles was dried on a rotary evaporator (Heidolph Hei-VAP precision) for 15 min at 20 mbar. The pellet was dissolved in dichloromethane (DCM) and stirred at room temperature to break the nanoparticles and release the peptide. Subsequently, DCM was eliminated at reduced pressure and the released peptide was re-suspended in 25 mM phosphate buffer, pH 7.0 [46]. In the indirect method, the amount of peptide that was not encapsulated during the nanoparticle purification was quantified. The peptide collected by both methods was quantified by reverse phase high efficiency liquid chromatography (RP-HPLC) using a C18 column, equilibrated with two solvent systems: 30% (A) H_2_O type 1 with 0.01% TFA and 70% (B) ACN with 0.01% TFA in runs of 15 min using an ultraviolet detector (DAD) at a wavelength of 280 nm [47]. Alternatively, the peptide was quantified by spectrophotometry at λ = 28 0nm, recorded in a UV-VIS spectrophotometer (Shimadzu UV-1800) and compared with a calibration curve of the peptide dissolved in 25 mM phosphate buffer, pH 7.0 [46]. The encapsulation efficiency (Equation (1)) was defined as the ratio between the amount of encapsulated peptide and the total amount of the peptide loaded for the encapsulation [48].

(1) $$\mathrm{Encapsulation}\;\mathrm{Efficency}=\frac{\mathrm{Encapsulated}\;\mathrm{peptide}\;\left(µg\right)}{\mathrm{Total}\;\mathrm{loaded}\;\mathrm{peptide}\;\left(µg\right)}\times 100$$

Moreover, in order to determine the maximum loading capacity of PLGA, AMP encapsulations were performed using different µg peptide/mg PLGA ratios (ranging from 0.125 to 16 μg/mg), determining encapsulated peptide as described previously. Peptide loading was calculated using as the ratio of amount encapsulated antimicrobial peptide and PLGA polymer (Equation (2)). All the tests were carried out by triplicate (*n* = 3).

(2) $$Peptide\;loading=\frac{Encapsulated\;peptide\;\left(µg\right)}{PLGA\;total\;\left(\mathrm{mg}\right)}$$

### 3.6. In Vitro Release of Peptide from Nps

Ten Eppendorf tubes with 1 mL of nanoparticles synthesized with a ratio of 8 μg AMP per mg PLGA, were dispersed in 25 mM buffer phosphate, pH 7.0 and Eppendorf test tubes, these tubes were incubated at 37 °C and shaken at 80 rpm in an orbital shaker (Thermo Scientific MaxQ 4000). At different times, the peptide released in the Eppendorf was recovered by centrifugation using an Amicon of 30,000 MW ultrafilter separated by RP-HPLC and quantified as described above.

### 3.7. Determination of Minimum Inhibitory Concentration (MIC) and Minimum Bactericidal Concentration (MBC)

The antimicrobial activity of free and encapsulated peptide was evaluated by the broth micro dilution method described in previous works [18,49]. Briefly, a pre-inoculum of *E. coli* O157:H7 in LB and MRSA in MH were grown at 37 °C for 12 h at 200 rpm; then, the culture of each strain was set at 0.5 in a McFarland scale (10^8^ CFU ml^−1^); 100 μL aliquots of these cell suspensions were mixed with 100 μL of AMP-NPs or empty NP solutions at different concentrations in a 96 well microplate and incubated at 37 °C in an orbital shaker (200 rpm for 8 h). The bacterial growth kinetics of these microbial cultures was performed by measuring changes of absorbance at 595 nm over time in an Elisa reader for these cultures (Bio-Rad, iMark). The controls were: (1) culture media, (2) culture media plus bacteria, (3) culture media, bacteria and ofloxacin and (4) culture media, bacteria and empty NPs. MIC_50_ was defined as the lowest concentration of AMP-NPs inhibiting 50% of the bacterial growth of these bacterial strains. After incubation for 8 h, 100 μL of these bacterial cultures were poured in 900 μL of BHI, incubated at 37 °C for 24 h; then, 10 μL of these cultures were seeded over BHI-agar petri dishes, incubated for 1 day at 37 °C and the appearance of colonies were determined. The MBC was the lowest nanoparticle concentration producing >99.9% reduction of colony-forming units (CFU).

### 3.8. Statistical Analysis

Normalized data were expressed as the mean ± SEM and analyzed using the Statistica software (StatSoft Inc., Tulsa, OK, USA). The analysis of variance (one-way ANOVA) and Student’s t-test comparisons were used to assess statistical significance at *p* < 0.05.

## 4. Conclusions

In this work, it was possible to design a new peptide (G19) that demonstrated strong antibacterial activity against *E. coli* O157:H7 and MRSA (values of MIC 50 in ranges of 12.5 and 1.5 μM, respectively). Peptide G19 presented a higher antimicrobial activity against MRSA than the previously published G17. This makes the two peptides more powerful than natural peptides such as LL-37, plectasin, or nisin.

There are multiple challenges regarding the design and incorporation of antimicrobial peptides into nanoparticulate systems. This work explored a new encapsulation methodology for both peptides that differs from the one evaluated for G17 in Cruz et al. This new methodology allows us to improve the loading capacity and the antimicrobial activity (MIC50) of the peptides encapsulated by 2–4 times. The improvement in the antimicrobial activity must be related to the increase in the local peptide concentration at the bacterial surface. 

Finally, the new G19 antimicrobial peptide has great antimicrobial potential, which can be improved through nanoencapsulation strategies, with an efficient and gradual release of the active compound. The peptides evaluated here make good candidates for further in vivo studies. 

## Figures and Tables

**Table 1:** Physicochemical properties of peptide G17 and G19 calculated using ExPASy ProtParam tool and CAMP R3 software.

Peptide	Sequence	Net Charge	MW (Da)	m/z	PAP (%)	pI	II	GRAVY
*G17*	ATKKCGLFKILKGVGKI	5	1804.31	1804.19	96	10.20	18.06	0.382
*G19*	ATKKCALWSILKAVAKI	4	1844.33	1864.19	97.3	10.04	24.68	0.735

**MW**: Molecular Weight; **PAP**: Predict Antimicrobial Peptides; **pI:** Isoelectric Point; **II:** Instability Index; **GRAVY:** Grand Average of Hydropathy.

**Table 2:** Physicochemical properties of different AMP-NPs at different peptide/polymer ratios.

NP	(µg AMP /mgPLGA) Ratio	Mean Zeta Potential (mV)			Mean Size (nm)			Mean PDI			Mean Encapsulation Efficiency (%)			Mean Peptide Loading (µg AMP/mg PLGA)		
*Empty NP*	0	−21.97	±	1.25	270.87	±	2.96	0.11	±	0.03	-		-	-		-
*G17NP*	16	8.56	±	0.30	293.20	±	1.61	0.24	±	0.03	49.50	±	0.70	7.92	±	0.11
	8	7.26	±	1.11	284.50	±	3.61	0.22	±	0.02	90.59	±	0.67	7.25	±	0.05
	4	3.99	±	0.23	303.33	±	18.65	0.27	±	0.07	76.10	±	1.87	3.04	±	0.07
	2	−2.06	±	0.21	1022.40	±	317.18	0.83	±	0.27	67.58	±	0.83	1.35	±	0.02
	1	−14.10	±	0.56	322.27	±	4.25	0.22	±	0.02	83.79	±	0.14	0.84	±	0.00
	0.50	−19.03	±	0.64	339.53	±	1.86	0.12	±	0.08	86.19	±	3.59	0.43	±	0.02
	0.25	−20.80	±	0.46	285.37	±	1.61	0.08	±	0.04	87.64	±	3.66	0.22	±	0.01
	0.12	−23.37	±	0.45	266.30	±	4.35	0.12	±	0.04	88.48	±	3.01	0.11	±	0.00
*G19NP*	16	12.90	±	0.60	291.67	±	3.38	0.16	±	0.11	50.85	±	1.20	8.14	±	0.19
	8	12.87	±	1.02	291.77	±	1.59	0.26	±	0.06	90.60	±	0.94	7.25	±	0.07
	4	12.90	±	0.80	257.83	±	2.84	0.13	±	0.05	80.48	±	1.76	3.22	±	0.07
	2	3.03	±	0.30	290.70	±	3.67	0.08	±	0.04	79.27	±	2.26	1.59	±	0.04
	1	−1.86	±	0.37	432.63	±	6.66	0.16	±	0.04	51.10	±	4.98	0.51	±	0.05
	0.50	−11.80	±	0.95	1976.00	±	496.56	0.82	±	0.12	41.05	±	3.18	0.21	±	0.02
	0.25	−15.77	±	0.40	353.67	±	5.93	0.17	±	0.02	89.92	±	6.88	0.22	±	0.02
	0.12	−18.73	±	0.40	324.87	±	7.27	0.12	±	0.05	89.45	±	4.39	0.11	±	0.01

AMP-NP: Encapsulated Antimicrobial peptide; AMP: Antimicrobial peptide; PLGA: Poly-lactic-glycolic-acid; PDI: poly dispersion index.

**Table 3:** Antimicrobial activity of free and encapsulated G17 and G19.

*Microorganism*	*E. coli* O157:H7			MRSA		
*Compound*	MIC_50_ (µM)	MIC_90_ (µM)	MBC (µM)	MIC_50_ (µM)	MIC_90_ (µM)	MBC (µM)
*G17*	12.5	20	70	1.5	12.5	100
*G17NP*	3.13	25	>100	0.2	12.5	25
*G19*	12.5	20	70	1.5	6.5	70
*G19NP*	3.13	20	100	0.7	20	100
*NP*	>100	>100	>100	>100	>100	>100

## Supplementary Materials

The following are available online at https://www.mdpi.com/2079-6382/9/7/384/s1, Figure S1: MALDI-ToF mass spectra of G17 (**A**) and G19 (**B**) the samples were prepared using a matrix of α-cyano-4-hydroxycinnamic acid (CHCA), the peptide was deposited on the target by the double layer method, that is, a saturated matrix base, followed by the sample and finally a second matrix layer, Figure S2: Circular dichroism spectra of G17 (**A**) and G19 (**B**) under simulated membrane conditions in 30% (*v/v*) trifluoroethyl alcohol (TFE) solution.
