# Supplementary material for: Potent and Specific Antibacterial Activity against Escherichia coli O157:H7 and Methicillin Resistant Staphylococcus aureus (MRSA) of G17 and G19 Peptides Encapsulated into Poly-Lactic-Co-Glycolic Acid (PLGA) Nanoparticles
Source: Antibiotics (Basel). 2020 Jul 7;9(7):384. doi: 10.3390/antibiotics9070384 (PMC7400247; doi:10.3390/antibiotics9070384)
Supplement: Supplementary file 1 [file antibiotics-09-00384-s001.pdf]

SUPPLEMENTARY MATERIALS

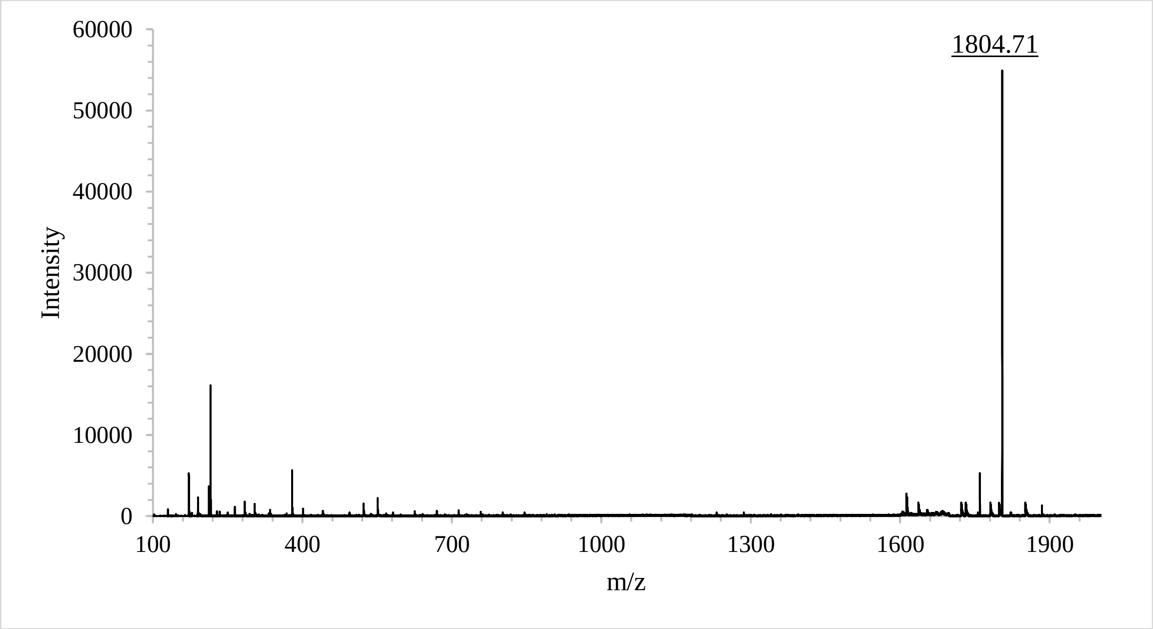

(A)

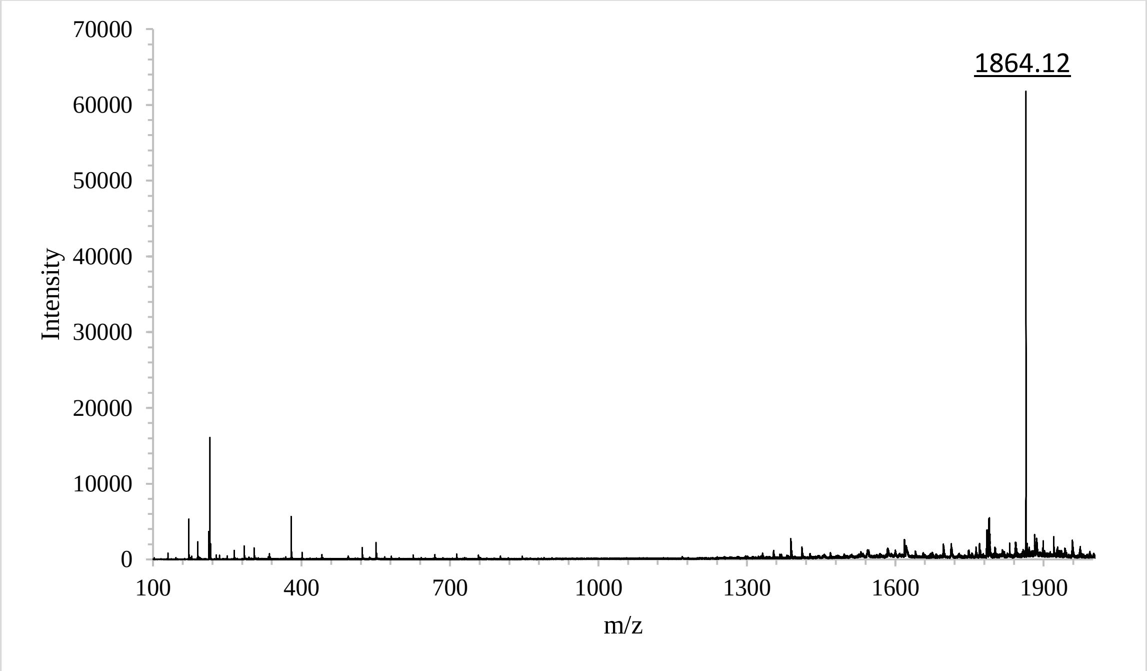

(B)

Figure S1. MALDI-ToF mass spectra of G17 (A) and G19 (B), the samples were prepared using a matrix of α-cyano-4-hydroxycinnamic acid (CHCA), the peptide was deposited on the target by the double layer method, that is, a saturated matrix base, followed by the sample and finally a second matrix layer

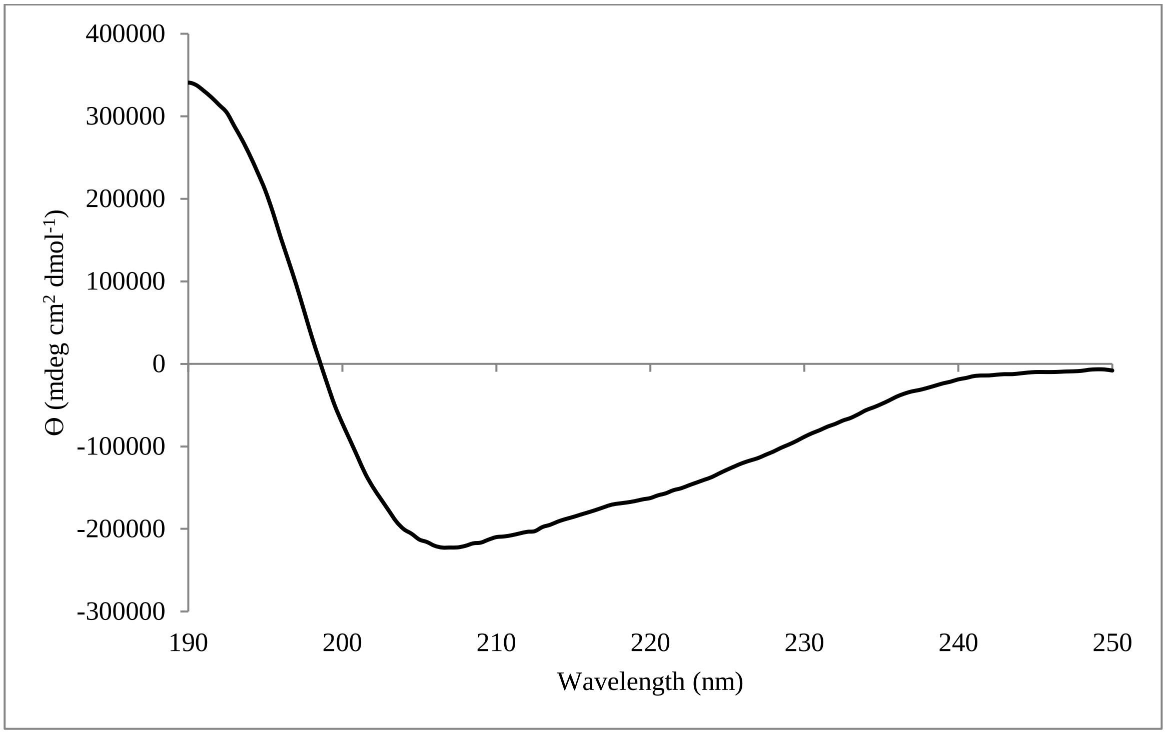

(A)

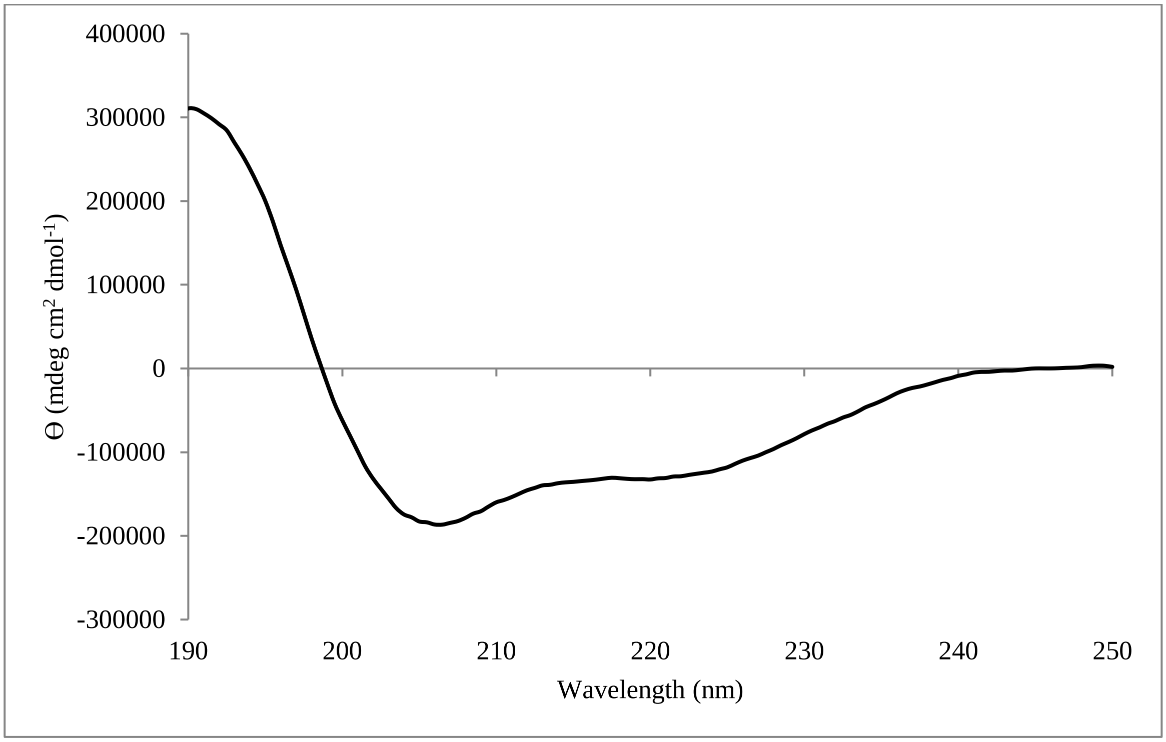

(B)

Figure S2. Circular dichroism spectra of G17 (A) and G19 (B) under simulated membrane conditions in 30% (v/v) trifluoroethyl alcohol (TFE) solution.
